# The two roles of complex III in plants

**DOI:** 10.7554/eLife.65239

**Published:** 2021-01-19

**Authors:** Hans-Peter Braun

**Affiliations:** Institut für Pflanzengenetik, Leibniz Universität HannoverHannoverGermany

**Keywords:** vigna radiata, mitochondria, respiration, membrane protein, cryoEM, supercomplex, enzymes, Other

## Abstract

Atomic structures of mitochondrial enzyme complexes in plants are shedding light on their multiple functions.

**Related research article** Maldonado M, Guo F, Letts JA. 2021. Atomic structures of respiratory complex III_2_, complex IV and supercomplex III_2_-IV from vascular plants. *eLife* **10**:e62047. doi: 10.7554/eLife.62047

Every year land plants assimilate about 120 billion tons of carbon from the atmosphere through photosynthesis (Jung et al., 2011). However, plants also rely on respiration to produce energy, and this puts about half the amount of carbon back into the atmosphere (Gonzalez-Meler et al., 2004). Mitochondria have a central role in cellular respiration in plants and other eukaryotes, harboring the enzymes involved in the citric acid cycle and the respiratory electron transport chain.

The basic functioning of mitochondria is highly conserved across evolution. In plants, however, these organelles perform additional roles linked to photosynthesis (Millar et al., 2011). In particular, under certain conditions they employ enzymes called alternative oxidoreductases, which may help to reduce the formation of reactive oxygen species (Vanlerberghe, 2013).

The mitochondrial electron transport chain is similar in plants, fungi and animals, where it is formed of four enzyme complexes – complex I, II, III and IV – as well as further components such as cytochrome c and the lipid ubiquinone. In plants, alternative oxidoreductases are also involved. The structure and function of the complexes I to IV have been extensively investigated in animals and fungi, but less so in plants. Now, in eLife, Maria Maldonado, Fei Guo and James Letts from the University of California Davis present the first atomic models of the complexes III and IV from plants, giving astonishing insights into how the mitochondrial electron transport chain works in these organisms (Maldonado et al., 2021).

For their investigation, Maldonado et al. isolated mitochondria from etiolated mung bean seedlings; the protein complexes of the electron transport chain were then purified, and their structure was analyzed using a new experimental strategy based on single-particle cryo-electron microscopy combined with computer-based image processing (Kuhlbrandt, 2014). The team used pictures of 190,000 complex I particles, 48,000 complex III_2_ particles (III_2_ is the dimer formed by complex III), and 28,000 particles of a supercomplex consisting of complexes III_2_ and IV. Average structures of all three types of particles were calculated with resolutions in the range of 3.5 Angstroms, which allow side chains of amino acids to become visible. Finally, the amino acid sequences of the protein subunits were fitted into the structures, generating models of the protein complex at atomic resolution. Results of this experimental approach on a large subcomplex of plant complex I have been published before, and they revealed an extra functional module that may be relevant when cellular respiration takes place alongside photosynthesis (Maldonado et al., 2020). This suggests that complex I has additional roles in plants.

Now, the atomic models for plant complex III_2_ and the supercomplex III_2_-IV indicate that these share highly conserved features with the corresponding animal and fungi structures. The plant complex IV, for instance, which had never been precisely defined so far, consists of 10 protein subunits that are all homologs of animal and fungi complex IV subunits. Similarly, plant complex III_2_, which is formed of a pair of 10 subunits, much resembles its animal and fungi counterparts, and performs a similar role in cellular respiration (Figure 1).

**Figure 1.**
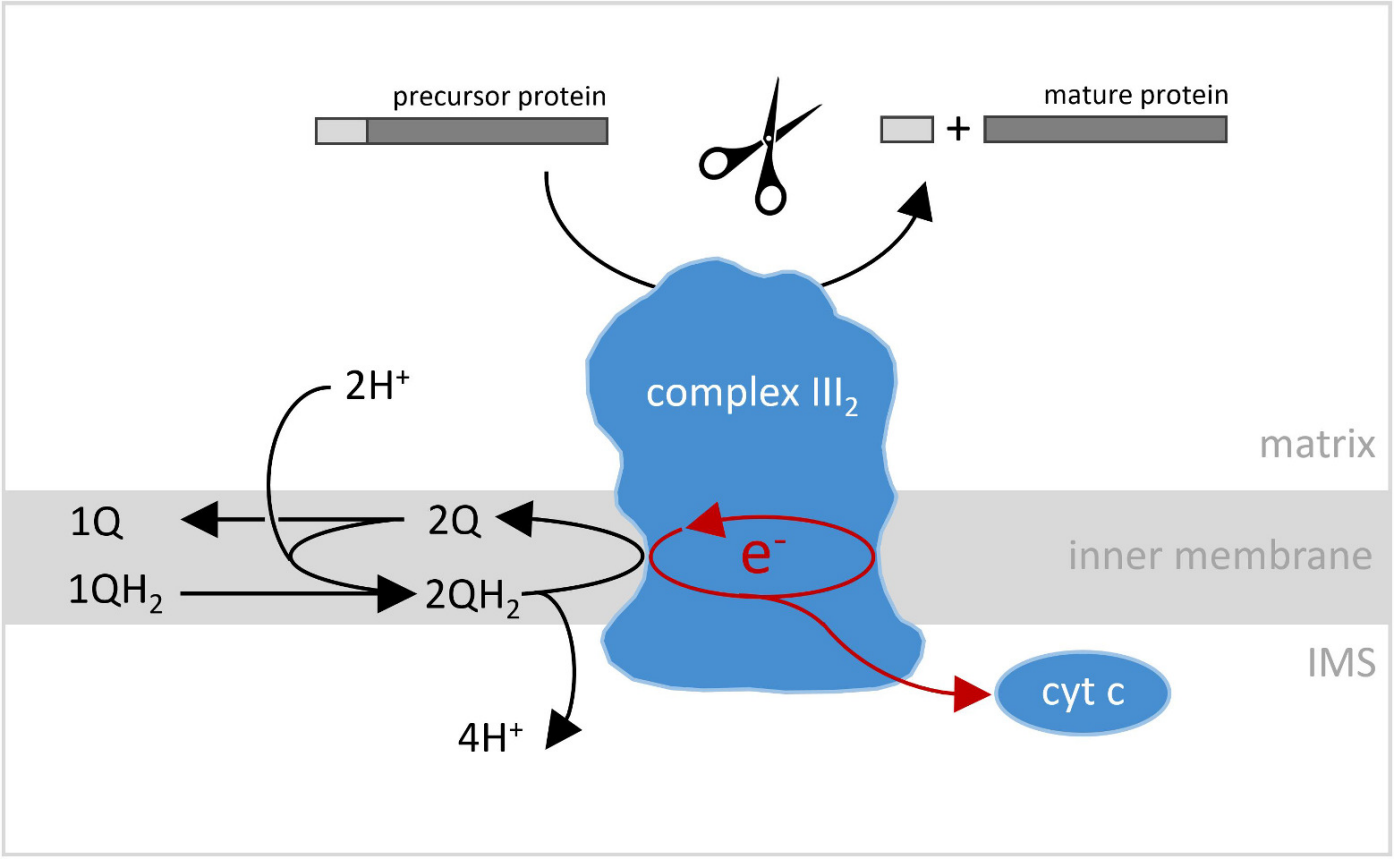
Reactions catalyzed by complex III_2_ in plant mitochondria. Complex III_2_ is an enzyme complex embedded in the inner membrane (light gray band) that separates the matrix (top) and the inter-membrane space (IMS; bottom) in mitochondria. One of the reactions it catalyzes is an oxidoreduction, where two molecules of ubiquinol (QH_2_) are sequentially converted into ubiquinone (Q), two pairs of protons are released into the mitochondrial intermembrane space (IMS), and two pairs of electrons are transferred onto complex III_2_. Two of these electrons are sequentially transferred onto cytochrome c (cyt c), while the other two electrons are used to convert one molecule ubiquinone into ubiquinol. The latter step is linked to the uptake of two protons from the mitochondrial matrix. The overall reaction results in protons being translocated across the inner mitochondrial membrane, which is a key process in cellular respiration. However, in plants, complex III_2_ is also involved in another reaction: it helps mitochondrial preproteins mature by removing the import sequence necessary for their movement into the compartment (top).

Image credit: Shape of the complex III_2_ is adapted from Maldonado et al., 2021.

However, at the same time, complex III_2_ is special in plants because it also includes the activity of the mitochondrial processing peptidase (MPP for short), an enzyme which cleaves off the transit sequences that help to import certain preproteins into mitochondria (Braun et al., 1992; Eriksson et al., 1994; Figure 1). For the very first time, structural details of the two MPP subunits of plant complex III_2_ are presented, showing that they form a large central cavity with a negative surface that is probably essential for preprotein binding. The structural model also includes a catalytic zinc ion at the active site of the subunit which cleaves the preproteins. In addition, compared to similar protein subunits in animal or fungi complex III_2_, the plant MPP subunits are more stably connected to the remaining complex III_2_ subunits. Finally, Maldonado et al. offer further structural insights into the functioning of complex IV and supercomplex III_2_-IV, which provide clues as to the way their enzyme activities take place.

Overall, the bifunctionality of complex III_2_ in plants may be another example that mitochondria work differently in the context of photosynthesis. Indeed, the way MPP is attached to complex III reflects that the presence of chloroplasts makes it more complicated for proteins to be transported and processed within plant cells. The atomic models revealed by Maldonado et al. will help further genetic and biochemical investigations into the physiology of the mitochondrial electron transport chain of plants.

## References

[bib1] Braun HP, Emmermann M, Kruft V, Schmitz UK (1992). The general mitochondrial processing peptidase from potato is an integral part of cytochrome c reductase of the respiratory chain. The EMBO Journal.

[bib2] Eriksson A, Sjöling S, Glaser E (1994). The ubiquinol cytochrome c oxidoreductase complex of spinach leaf mitochondria is involved in both respiration and protein processing. Biochimica Et Biophysica Acta (BBA) - Bioenergetics.

[bib3] Gonzalez-Meler MA, Taneva L, Trueman RJ (2004). Plant respiration and elevated atmospheric CO_2_ concentration: cellular responses and global significance. Annals of Botany.

[bib4] Jung M, Reichstein M, Margolis HA, Cescatti A, Richardson AD, Arain MA, Arneth A, Bernhofer C, Bonal D, Chen J, Gianelle D, Gobron N, Kiely G, Kutsch W, Lasslop G, Law BE, Lindroth A, Merbold L, Montagnani L, Moors EJ, Papale D, Sottocornola M, Vaccari F, Williams C (2011). Global patterns of land-atmosphere fluxes of carbon dioxide, latent heat, and sensible heat derived from eddy covariance, satellite, and meteorological observations. Journal of Geophysical Research.

[bib5] Kuhlbrandt W (2014). The resolution revolution. Science.

[bib6] Maldonado M, Padavannil A, Zhou L, Guo F, Letts JA (2020). Atomic structure of a mitochondrial complex I intermediate from vascular plants. eLife.

[bib7] Maldonado M, Guo F, Letts JA (2021). Atomic structures of respiratory complex III_2_, complex IV and supercomplex III_2_-IV from vascular plants. eLife.

[bib8] Millar AH, Whelan J, Soole KL, Day DA (2011). Organization and regulation of mitochondrial respiration in plants. Annual Review of Plant Biology.

[bib9] Vanlerberghe GC (2013). Alternative oxidase: A mitochondrial respiratory pathway to maintain metabolic and signaling homeostasis during abiotic and biotic stress in plants. International Journal of Molecular Sciences.

